# A Process × Domain Assessment of Narcissism: The Domain-Specific Narcissistic Admiration and Rivalry Questionnaire

**DOI:** 10.1177/10731911211020075

**Published:** 2021-06-04

**Authors:** Michael P. Grosz, Isabel Hartmann, Michael Dufner, Marius Leckelt, Tanja M. Gerlach, John F. Rauthmann, Jaap J. A. Denissen, Albrecht C. P. Küfner, Mitja D. Back

**Affiliations:** 1 University of Münster, Münster, Germany; 2 Witten/Herdecke University, Witten, Germany; 3 Leipzig University, Leipzig, Germany; 4 University of Mainz, Mainz, Germany; 5 University of Göttingen, Göttingen, Germany; 6 Bielefeld University, Bielefeld, Germany; 7 Utrecht University, Utrecht, Netherlands

**Keywords:** narcissism, intelligence, physical attractiveness, social status, communion

## Abstract

Research on grandiose narcissism distinguishes between self-promotional processes (i.e., *narcissistic admiration*) and other-derogative processes (i.e., *narcissistic rivalry*; Back et al., 2013). Moreover, research has begun to assess and investigate narcissistic manifestations in different domains (e.g., communal narcissism). To integrate these two lines of research, we developed the *Domain-Specific Narcissistic Admiration and Rivalry Questionnaire* (D-NARQ), a 72-item narcissism questionnaire that contains a self-promotional process scale (narcissistic admiration) and an other-derogatory process scale (narcissistic rivalry) for four domains: intellectual ability, social dominance, communal care, and physical attractiveness. We investigated the psychometric properties of the D-NARQ in a large online study (*N* = 1,635). Model fit statistics were largely in line with the theorized factor structure. The D-NARQ scales had good to very good measurement precision, and their correlations with established narcissism scales, the Big Five personality traits, and comparative self-evaluations largely supported their convergent and discriminant validity.

Grandiose narcissism is a multifaceted personality trait characterized by striving for a grandiose self and superiority over others. To understand the processes behind narcissistic strivings (i.e., how narcissism is acted out), Back et al. (2013) developed the Narcissistic Admiration and Rivalry Concept (NARC). It distinguishes self-promotional processes (i.e., *narcissistic admiration*) from other-derogative processes (i.e., *narcissistic rivalry*). Narcissistic admiration is characterized by the tendency to enhance the positivity of one’s self-views and to employ assertiveness and charm to garner admiration from others. Narcissistic rivalry is characterized by the tendencies to employ antagonism and devaluate others to protect oneself from negative self-views. A large body of research has shown that narcissistic admiration and narcissistic rivalry are distinct narcissism dimensions that are differentially related to a range of intra- and interpersonal behaviors, characteristics, and outcomes (for an overview, see Back, 2018). In fact, admiration and rivalry even exhibit opposing associations with a number of constructs, such as self-esteem (Geukes et al., 2017), popularity (Leckelt et al., 2015), and attractiveness as a mate (Wurst et al., 2017).

In a separate line of research, different domains of grandiose narcissism have been investigated. These domains are playing fields on which narcissism is acted out, and the respective domain determines how narcissistic strivings are expressed and fulfilled. For example, Gebauer, Sedikides, et al. (2012) proposed the agency–communion model of narcissism in which the trait of narcissism can be acted out in the agency domain or in the communion domain. In the agency domain, narcissism is expressed through agentic means, for instance, via grandiose self-promotive thoughts about the agentic traits assertiveness (“I am assertive”) and competence (“I am more capable than other people”; Gebauer, Sedikides, et al., 2012; see also Campbell & Foster, 2007). In the communion domain, narcissism is expressed through communal means, for instance, via grandiose self-promotive thoughts about the communal traits helpfulness (“I am the most helpful person”) and trustworthiness (“I am extraordinarily trustworthy”; Gebauer, Sedikides, et al., 2012). This line of research indicates that it is expedient to assess not only general (domain-unspecific) forms of narcissism but also domain-specific forms of broad narcissistic traits because agentic narcissism (as measured with the Narcissistic Personality Inventory [NPI]; Raskin & Hall, 1979) and communal narcissism (as measured with the Communal Narcissism Inventory [CNI]; e.g., Gebauer, Sedikides, et al., 2012) have been found to be only moderately correlated with one another (*r* = .27; Gebauer, Sedikides, et al., 2012). Furthermore, both agentic and communal narcissism have shown a high level of temporal stability (8-week test–retest reliabilities of *r* = .71 and .79, respectively) and have been found to be related differently to self-perceptions (e.g., self-perceived prosociality) and actual behavior (e.g., prosocial behavior; agentic and communal overclaiming; e.g., Gebauer, Sedikides, et al., 2012; Nehrlich et al., 2019). Other domain-specific forms of narcissism (e.g., sexual narcissism) have been introduced as well (e.g., Widman & McNulty, 2010). Yet, a comprehensive approach for assessing different domain-specific forms of narcissism is still missing.

Despite the accumulating evidence of discriminant correlates and outcomes of process- and domain-specific narcissism measures, previous research on self-promotional and other-derogative processes has not differentiated between domains, and research on domain-specific narcissism has not differentiated between self-promotional and other-derogative processes. Consequently, narcissism measures unsystematically capture different processes and domains across items (e.g., NPI; Raskin & Hall, 1979), include only domain-unspecific items for self-promotion and other-derogation processes (e.g., the Narcissistic Admiration and Rivalry Questionnaire, NARQ; Back et al., 2013), or focus on specific process × domain combinations only (e.g., CNI; Gebauer, Sedikides, et al., 2012). Table 1 provides an illustrative overview of the domains that the existing grandiose narcissism items capture in terms of our process × domain conceptualization.

**Table 1.** Sorting the Narcissism Items According to the Process × Domain Conceptualization of Narcissism.

		Intellectual ability	Social dominance	Communal care	Physical attractiveness	Domain-unspecific
Self-promotion (narcissistic admiration)	NPI	—	I have a natural talent for influencing people.	—	I like to display my body.	I am an extraordinary person.
	NGS	—	High-status	—	—	Superior
	FFNI	—	Leadership comes easy for me.	—	I like to wear clothing that is considered trendy and fashionable.	I am a superior person.
	CNI	—	—	I am the most helpful person I know.	—	—
	NARQ	—	—	—	—	I am great.
	D-NARQ	I am a genius.	I am very assertive.	I am extraordinarily helpful.	I am very good looking.	—
Other-derogation (narcissistic rivalry)	NPI	—	I insist on getting the respect that is due me.	—	I get upset when people don’t notice how I look when I go out in public.	I will never be satisfied until I get all that I deserve.
	NGS	—	—	—	—	—
	FFNI	—	I do not get along with people who question my authority.	—	—	I do not waste my time hanging out with people who are beneath me.
	CNI	—	—	—	—	—
	NARQ	—	—	—	—	Most people are somehow losers.
	D-NARQ	Most people are stupid.	Most people are weaklings.	Most people are reckless egoists.	Most people are not particularly attractive.	—

*Note*. NPI = Narcissistic Personality Inventory (Raskin & Hall, 1979); NGS = Narcissistic Grandiosity Scale (Crowe et al., 2016; Rosenthal et al., 2020); FFNI = Five Factor Narcissism Inventory (Glover et al., 2012); CNI = Communal Narcissism Inventory (Gebauer, Sedikides, et al., 2012); NARQ = Narcissistic Admiration and Rivalry Questionnaire (Back et al., 2013); D-NARQ = Domain-Specific Narcissistic Admiration and Rivalry Questionnaire.

The current study brings process- and domain-specific narcissism research together by proposing that the two processes of narcissistic admiration and rivalry can be acted out in at least four different domains: (a) intellectual ability, (b) social dominance, (c) communal care, and (d) physical attractiveness. In each of these four domains, the two narcissistic processes can be expressed via domain-specific thoughts, feelings, and behaviors that are self-promoting and other-derogative, respectively. In the intellectual ability domain, narcissistic admiration and rivalry can be expressed, for example, via grandiose self-promotive thoughts about one’s own intellectual ability (e.g., “I am extraordinarily intelligent”; “I am a genius”), and other-derogative thoughts about others’ intellectual ability (e.g., “most people are stupid”; “others do not deserve to be admired for their intellectual abilities”). Similar self-promotive and other-derogative thoughts can be expected in the other three domains (see Table 1 for examples). Domain-specific forms of narcissistic admiration and rivalry should be related not only to domain-specific thoughts but also to domain-specific behavioral reactions to domain-specific ego-boosting opportunities and ego threats, as indicated by the NARC (Back et al., 2013).

In choosing these four domains (intellectual ability, social dominance, communal care, and physical attractiveness), we aimed to capture basic facets of social perception. Following an integrated framework for social evaluations (Abele et al., 2021), intellectual ability and social dominance mirror the ability and assertiveness facets of agency, respectively, whereas communal care taps into the morality and friendliness facets of communion. Physical attractiveness is another very powerful aspect of social evaluations, particularly when romantic relationship contexts are considered (e.g., Fletcher et al., 2004; Gebauer, Leary, et al., 2012). All four selected domains have been found to be relevant in narcissism research. The intellectual ability domain is relevant because many people high on narcissism consider intelligence to be a crucial means for attaining narcissistic goals, hold overly positive intellectual self-views, and are strongly motivated to appear intelligent to other people (for a review, see Zajenkowski & Dufner, 2020). The social dominance domain is relevant because social status and leadership are central goals for many people high in narcissism (e.g., Grapsas et al., 2020; Zeigler-Hill et al., 2019). The communal care domain is relevant because previous research indicates that some individuals strive for narcissistic goals in the communal domain (e.g., Gebauer, Sedikides, et al., 2012). Finally, the physical attractiveness domain is relevant because many people high in narcissism care strongly about their appearance and about being attractive to potential romantic partners (e.g., Holtzman & Strube, 2013).

Although these four domains are not the only potentially relevant playing fields in which narcissism is acted out, they should suffice for (a) a proof-of-concept of the integration of process-specific and domain-specific narcissism research and (b) a basis for developing a questionnaire that allows key narcissistic processes and domains to be differentiated. We named the new questionnaire the *Domain-Specific Narcissistic Admiration and Rivalry Questionnaire* (D-NARQ). The D-NARQ and its 2 (processes) × 4 (domains) approach should extend the ability to provide a specific and systematic conceptualization and assessment of narcissism. In addition, our process × domain approach may assist in classifying established narcissism measures according to process and domain content (Table 1).

All process- and domain-specific forms of narcissism share the striving for a grandiose self and superiority over others. It seems likely that a person with strong such strivings will act them out not only via one process or in one distinct domain but often via both processes and in several domains. Thus, all eight process- and domain-specific forms of narcissism should be positively related to one another. At the same time, the process- and domain-specific forms of narcissism and their nomological networks should be somewhat distinct from one another for two reasons. First, narcissistic admiration and narcissistic rivalry are related but distinct process dimensions (e.g., Back et al., 2013). Hence, forms of narcissism characterized by the same process dimension (e.g., intellectual-ability-specific and social-dominance-specific narcissistic admiration) should be more strongly related to one another than forms of narcissism characterized by different processes (e.g., intellectual-ability-specific narcissistic admiration and social-dominance-specific narcissistic rivalry). Second, there should be individual differences in the propensities to act out narcissistic strivings in one domain rather than in others (for initial evidence, see, e.g., Gebauer, Sedikides, et al., 2012). The propensity to act out narcissistic admiration and rivalry in one domain rather than in others might be determined by the perceived opportunities for ego boosts and risks of ego threats in the various domains. For example, a person who is very intelligent but not very physically attractive might perceive that the intellectual ability domain offers more opportunities for ego boosts and less risk of ego threats than the physical attractiveness domain. Thus, this individual might develop intellectual-domain-specific narcissistic admiration and rivalry rather than physical-attractiveness-specific narcissistic admiration and rivalry. Hence, forms of narcissism in the same domain (e.g., narcissistic admiration and rivalry in the intellectual ability domain) should be more strongly related to one another than process-specific forms of narcissism in different domains (e.g., narcissistic admiration in the intellectual ability domain and narcissistic rivalry in the physical attractiveness domain). Furthermore, domain-specific forms of narcissism should be more strongly related to one another if the domains are closely related to one another (e.g., the two agentic domains of intellectual ability and social dominance) than if they are not closely related to one another (e.g., the agentic domain of intellectual ability and the communal domain of communal care).

## Method

### Participants and Procedure

We drew our data from an online survey that included personality and narcissism inventories. The participants were recruited via the Internet, campus advertisements, and e-mail lists. As an incentive, they received personality feedback and took part in a lottery for 6 × €50. The original sample comprised 1,682 German-speaking participants. Two participants were excluded because they responded very quickly (i.e., faster than 1 second per item; Wood et al., 2017). Twenty-one participants were excluded because they were multivariate outliers, as operationalized by a Mahalanobis distance of more than 3 standard deviations larger than the average Mahalanobis distance. Twenty-four participants were excluded due to invariant responding: They gave the same response on more than 34 consecutive narcissism items (i.e., this number was 3 standard deviations higher than the number of consecutive narcissism items answered by the average respondent with the same response). Of the remaining 1,635 participants, 73% were women. The average age was 27.3 years (*SD* = 8.3, range: 18-73). Participants’ first language was German for 92% of them, a combination of German and another language for 1%, Russian for 2%, Polish for 1%, and various other languages for the other participants.

We report how we determined the sample size, all data exclusions, and manipulations. We do not report all measures in the current manuscript because the complete data set contained over 600 variables pertaining to various narcissism and personality scales, an overclaiming questionnaire, and a variety of self-reported behaviors and background factors. The data have been used in several other articles (e.g., Back et al., 2013; Grosz et al., 2017; Grosz et al., 2019; Leckelt et al., 2018; Wetzel et al., 2016). None of these articles addressed the same research question or used the same set of variables as the current study. Only one other article used some of the D-NARQ items: Grosz et al. (2017) used 45 admiration items from the original item pool for the D-NARQ. The original item pool comprised 60 admiration and 60 rivalry items. Grosz et al. (2017) investigated the extents to which three domain-specific admiration scales Intellectual Ability, Social Dominance, and Physical Attractiveness (each measured with 15 items) and several other narcissism scales were correlated with overclaiming bias. The current study used 36 admiration and 36 rivalry items from the original item pool (eight scales with nine items each) to introduce the D-NARQ and investigate the psychometric properties of all eight D-NARQ scales. We did not preregister the current study.

### Measures

#### D-NARQ

Like the NARQ (Back et al., 2013), the D-NARQ was developed with the goal of assessing continuous narcissistic personality traits in the general population. The D-NARQ was not developed to diagnose or screen people for narcissistic personality disorder. Also like the NARQ, the D-NARQ focuses exclusively on grandiose narcissism and not on vulnerable narcissism. Item generation and selection were guided by the NARC (Back et al., 2013). The NARC states that each of the two process dimensions is characterized by three facets. For narcissistic admiration, these facets are grandiose fantasies (i.e., cognitive facet), striving for uniqueness (i.e., affective facet), and charmingness (i.e., behavioral facet). For narcissistic rivalry, these facets are devaluation of others (i.e., cognitive facet), striving for supremacy (i.e., affective facet), and aggressiveness (i.e., behavioral facet; for more information about each facet, see Back et al., 2013). While creating and selecting the items, the aim was to ensure that all three facets of each of the two process dimensions were adequately covered in each of the four domains (intellectual ability, social dominance, communal care, and physical attractiveness). To accomplish this aim, six of the authors of the current study developed a large item pool that contained several items for each facet–domain combination. Each author was responsible for one or several facet–domain combinations. The other authors reviewed and optimized each other’s items on the basis of their content validity and linguistic aspects. Afterward, the D-NARQ item pool comprised 120 items. Each of the four domains were captured by 30 items, 15 pertaining to narcissistic admiration and 15 to narcissistic rivalry. Two of the authors independently rated each of the 120 items from the original item pool in terms of conceptual fit on a scale ranging from 1 (*does not fit at all*) to 6 (*fits perfectly; M* = 4.13; *SD* = 1.48; two-way, consistency, average-measures ICC = .85). Conceptual fit was specified by the definitions of the facets, as provided in the article on the NARC (Back et al., 2013). For each domain, we selected the nine best-fitting admiration items and the nine best-fitting rivalry items, resulting in 72 D-NARQ items.

All 72 D-NARQ items were administered with a 6-point Likert-type response scale ranging from 1 (*do not agree at all*) to 6 (*agree completely*). Example items are presented in Table 1 (for the English and German versions of the 72 items, see Tables S1 to S4 available at https://osf.io/vqys9/).

#### NARQ

The NARQ is an 18-item measure of grandiose narcissism (Back et al., 2013). It assesses the two process dimensions (i.e., narcissistic admiration and rivalry) with nine domain-unspecific items each (Table 1). All NARQ items had to be answered on a 6-point Likert-type response scale.

#### NPI

The NPI (Raskin & Hall, 1979; Schütz et al., 2004) assesses grandiose narcissism with 40 forced-choice items. We used the total NPI scale score and the three NPI subscales identified by Ackerman et al. (2011): Leadership/Authority (L/A; 11 items), Grandiose Exhibitionism (GE; 10 items), and Entitlement/Exploitativeness (E/E; four items). In terms of our process × domain conceptualization, the L/A and GE subscales tend to capture self-promotional processes, whereas the E/E subscale tends to capture other-derogative processes. Regarding domains, L/A focuses on the social dominance domain and GE mostly focuses on the physical attractiveness domain. The NPI has also several domain-unspecific items (Table 1).

#### NGS

The Narcissistic Grandiosity Scale (NGS; Crowe et al., 2016; Rosenthal et al., 2020) is a measure of grandiose narcissism that asks respondents to rate themselves on adjectives. NGS items capture self-promotional processes mostly in the social dominance domain or are domain-unspecific (Table 1). The 16 NGS items were answered on a 7-point Likert-type response scale.

#### CNI

The CNI (Gebauer, Sedikides, et al., 2012) assesses a communal form of grandiose narcissism. In terms of our process × domain conceptualization, it captures self-promotion in the communal care domain (Table 1). The current survey included 11 of the 16 original CNI items, all of which had to be answered on a 7-point Likert-type response scale.

#### BFI-S

The Big Five personality traits were measured with a 15-item version of the Big Five Inventory (Gerlitz & Schupp, 2005; Hahn et al., 2012). It consists of three items for each Big Five trait, all of which had to be answered on a 7-point Likert-type response scale.

#### Comparative Self-Evaluations

We used 14 items to measure comparative self-evaluations in the four domains: intellectual ability (item content: “mental/academic abilities” and “good judgment”), social dominance (“leadership qualities” and “assertiveness”), communal care (“fidelity,” “helpfulness,” “hard-heartedness,” “honesty,” “unfriendliness,” “empathy,” “coldness,” “courtesy,” and “selfishness”), and physical attractiveness (“physical attractiveness”). Some of the items stemmed from the Self-Attributes Questionnaire (Pelham & Swann, 1989) and some from Schröder-Abé (2012). For each item, participants were asked to rate themselves in comparison with others on scales ranging from 1 (*bottom 5%*) to 10 (*top 5%*).

## Results

Most analyses were computed in R (version 4.0.2; R Core Team, 2018). The data, R code, and supplemental figures and tables can be found on the OSF project page at https://osf.io/vqys9/. For all significance tests, we used a significance level of .001 rather than .05 to reduce the likelihood of false positives and to prevent trivial effect sizes from reaching the level of significance. Means, medians, standard deviations, skewness, Cronbach’s alphas, McDonald’s omega total reliabilities, and gender differences for the scales were computed with the R package psych (version 2.0.9; Revelle, 2020) and are presented in Table S5. Interestingly, men scored significantly higher on all D-NARQ scales except for the narcissistic admiration scale from the physical attractiveness domain.

### Factor Structure

**Figure 1.**
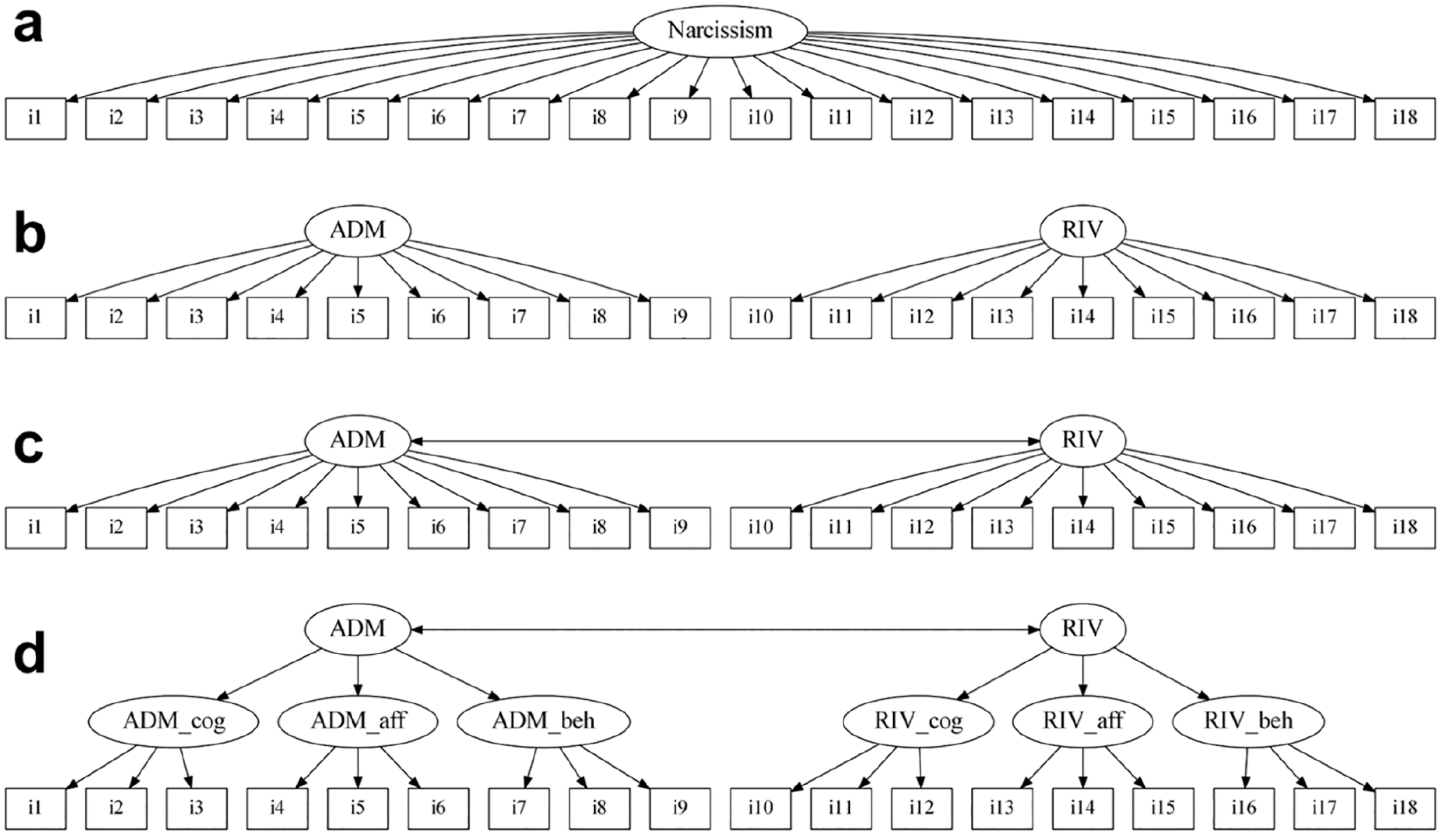
Confirmatory factor analysis models fit to the 18 D-NARQ items from each domain. *Note*. We fit (a) a one-factor model, (b) a two-factor model with two uncorrelated factors, (c) a two-factor model with two correlated factors, and (d) a second-order factor model to the 18 D-NARQ items from each of the four domains. The models in the intellectual ability, communal care, and physical attractiveness domains did not include six but only five or four first-order factors (for details, see Figures S1 to S3). ADM = narcissistic admiration; RIV = narcissistic rivalry; ADM_cog = cognitive facet of narcissistic admiration (i.e., grandiose fantasies); ADM_aff = affective facet of admiration (i.e., striving for uniqueness); ADM_beh = behavioral facet of admiration (i.e., charmingness); RIV_cog = cognitive facet of rivalry (i.e., devaluation of others); RIV_aff = affective facet of rivalry (i.e., striving for supremacy); RIV_beh = behavioral facet of rivalry (i.e., aggressiveness; Back et al., 2013); D-NARQ = Domain-Specific Narcissistic Admiration and Rivalry Questionnaire.

To investigate the factor structure, we fit four separate confirmatory factor analysis models to the 18 D-NARQ items in each domain: (a) a one-factor model, (b) a two-factor model with two uncorrelated factors, (c) a two-factor model with two correlated factors, and (d) a second-order factor model (see Figure 1). The four second-order factor models were identical to the second-order factor model that was fit to the NARQ in Back et al. (2013) with the exception that the models in the intellectual ability, communal care, and physical attractiveness domains did not include six but only five or four first-order factors (facets) because the variance of some of the first-order factors was negative in the model with six first-order factors. Whenever this was the case, the three items from the facet loaded directly on the second-order factor (for details, see Figures S1 to S3). We used the WLSMV estimator from the R package lavaan (version 0.6-6; Rosseel, 2012) because estimators based on least squares are recommended for ordinal data especially when items show floor effects as some D-NARQ items did (see the relatively low means and the skewness of some D-NARQ items in Tables S1 to S4; e.g., DiStefano & Morgan, 2014; Li, 2016; Savalei & Rhemtulla, 2013).

The scaled (robust) χ^2^ and scaled (robust) fit indices are presented in Table 2. In each domain, the second-order factor model showed the best χ^2^ and fit indices, a finding that was in line with the NARC (Back et al., 2013). We deemed the model fit of the four second-order models sufficient enough so that these theoretically informed models did not have to be rejected. The relatively lower comparative fit index in the communal care domain (.830) than in the other domains (.931 to .939) could be explained by the lower average standardized factor loadings among the communal care items (.66) than among the intellectual ability items (.79), social dominance items (.80), and physical attractiveness items (.82) given that the comparative fit index is influenced by the magnitude of the factor loadings (e.g., Moshagen & Auerswald, 2018). Furthermore, some of the less than optimal fit indices might have been due to overly restrictive assumptions of confirmatory factor analysis models, such as no cross-loadings (e.g., Marsh et al., 2010).

**Table 2.** Chi-Squares and Model Fits for Confirmatory Factor Analyses of the D-NARQ.

CFA model	*df*	χ^2^	CFI	RMSEA [90% CI]	SRMR
*Intellectual ability*					
One factor	135	5877.08	.793	.162 [.158, .165]	.118
Two factors (uncorrelated)	135	11693.29	.584	.229 [.226, .233]	.260
Two factors (correlated)	134	3777.13	.869	.129 [.126, .133]	.088
**Second-order factor model**	**129**	**2044.39**	**.931**	**.096 [.092, .099]**	**.066**
*Social dominance*					
One factor	135	6963.96	.811	.176 [.172, .180]	.148
Two factors (uncorrelated)	135	9789.21	.733	.209 [.206, .213]	.249
Two factors (correlated)	134	3654.14	.903	.127 [.123, .130]	.096
**Second-order factor model**	**128**	**2636.64**	**.931**	**.110 [.106, .113]**	**.079**
*Communal care*					
One factor	135	5772.52	.674	.160 [.157, .164]	.130
Two factors (uncorrelated)	135	5246.58	.704	.153 [.149, .156]	.160
Two factors (correlated)	134	3299.67	.817	.120 [.117, .124]	.097
**Second-order factor model**	**131**	**3069.62**	**.830**	**.117 [.114, .121]**	**.093**
*Physical attractiveness*					
One factor	135	7679.52	.810	.185 [.182, .189]	.149
Two factors (uncorrelated)	135	10008.18	.752	.212 [.208, .215]	.268
Two factors (correlated)	134	4163.39	.899	.136 [.132, .139]	.095
**Second-order factor model**	**130**	**2545.53**	**.939**	**.107 [.103, .110]**	**.074**

*Note.* We used confirmatory factor analyses with the WLSMV estimator in the R package lavaan (version 0.6-3; Rosseel, 2012). Bold font indicates the best fitting model according to χ^2^ and fit indices. For a graphical depiction of the models, see Figure 1. χ^2^ = scaled (robust) chi-square statistic; CFI = scaled (robust) comparative fit index; RMSEA = scaled (robust) root mean square error of approximation; CI = confidence interval; SRMR = scaled (robust) standardized root mean square residual; D-NARQ = Domain-Specific Narcissistic Admiration and Rivalry Questionnaire.

### Measurement Precision

To investigate measurement precision, we estimated test information curves for each of the eight D-NARQ scales by using graded response models (Samejima, 1968) and the R package mirt (version 1.33.2; Chalmers, 2012; for reliabilities, see Table S5 available online). The graded response model is a nonlinear item response theory model suitable for items with Likert-type response scales. Due to the nonlinearity of the model, the test information curve shows that the measurement precision is not equal across the latent trait continuum. The graded response models’ assumptions of unidimensionality and local independence were deemed plausible because, for each scale, the first factor explained at least 68% of the common variance when conducting a minimum rank factor analysis using the PC software package FACTOR (version 10.10.01; Lorenzo-Seva & Ferrando, 2006), and almost all residual item-pair correlations were below |.20| when testing a one-factor confirmatory factor analysis model using the R package lavaan (version 0.6-6; Rosseel, 2012). That is, across all eight scales, there were only eight residual item-pair correlations above |.20|, and all were smaller or equal to |.30| (for details, see Tables S6 to S13). Furthermore, we computed *S*-χ^2^ item fit statistics for the graded response models (e.g., Kang & Chen, 2011). Whenever there was significant item misfit, it was small in size (i.e., *r* ≤ .10; for details, see Tables S6 to S13).

**Figure 2.**
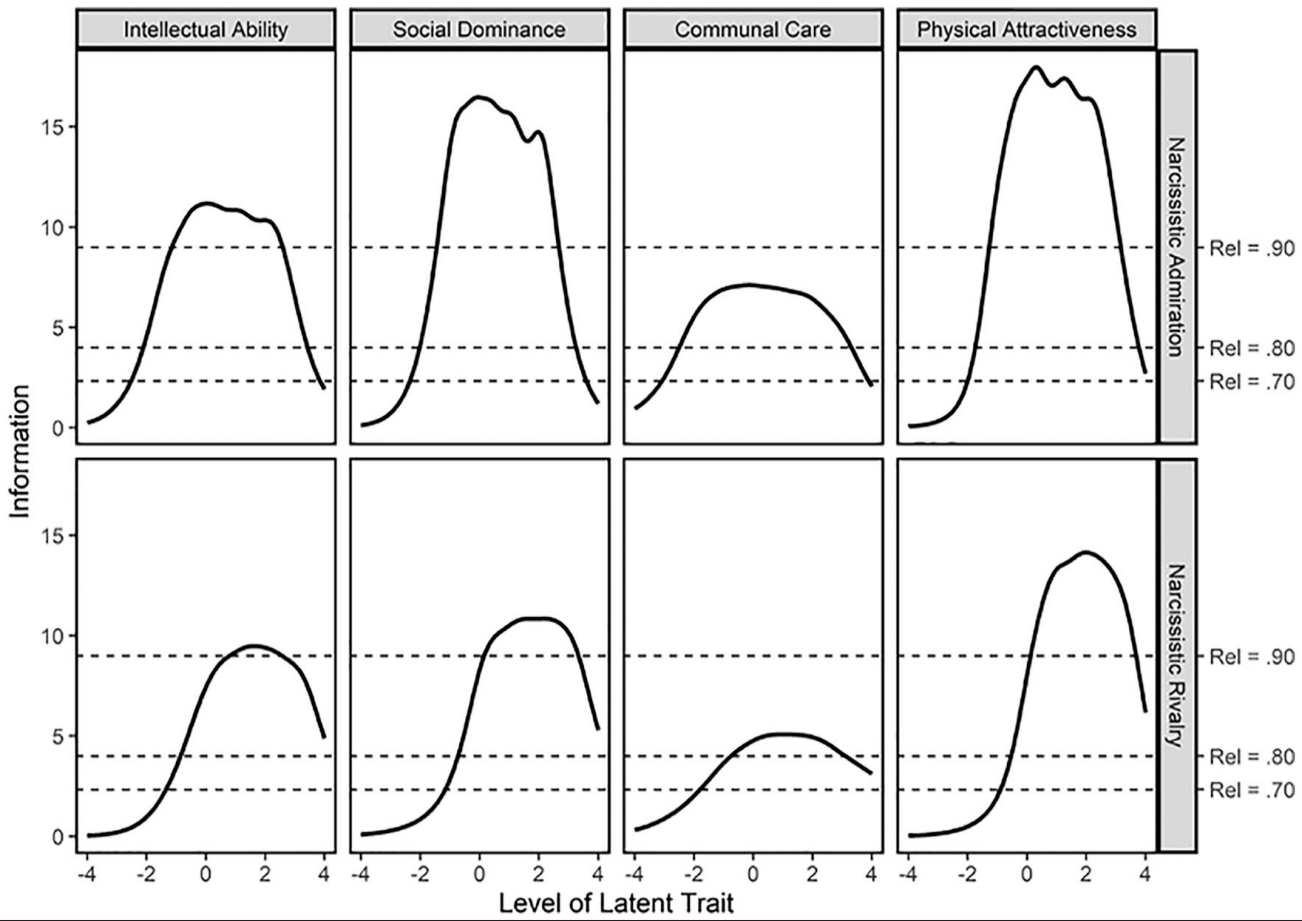
Test information as a function of latent trait level for the D-NARQ scales. *Note*. D-NARQ = The dashed lines represent reliability levels of .70, .80, and .90 under the assumption of a standard normally distributed latent trait in the population. In the latent trait range where the information curve exceeds these lines, the precision of the test score is comparable to that of a test for which the reliability is above .70, .80, and .90, respectively (for details, see Grosz et al., 2019; Samejima, 1994). D-NARQ = Domain-Specific Narcissistic Admiration and Rivalry Questionnaire.

As can be seen in the test information curves in Figure 2, all of the eight D-NARQ scales showed very high levels of measurement precision around the average and high levels of the latent trait continuum (between −1 to +4). The two communion care scales showed somewhat lower levels of measurement precision than the other scales, but the levels were still high. In the latent trait area between −2 and −1, most of the rivalry scales showed moderate measurement precision, whereas the admiration scales still showed high levels of precision. Taken together, although the D-NARQ rivalry scales could have been more precise in the low trait range, all the D-NARQ scales showed high levels of measurement precision across a broad range of their respective latent trait continuum.

### Intercorrelations Among the D-NARQ Scales

The intercorrelations among the eight D-NARQ scales are presented in Table 3. In line with the notion that all process- and domain-specific forms of narcissism share the striving for a grandiose self and superiority over others, all eight D-NARQ scales were positively correlated with one another. Because narcissistic admiration and rivalry are distinct process dimensions, forms of narcissism characterized by the same process dimension should be more strongly related to one another than forms of narcissism characterized by different process dimensions. In line with this notion, most of the D-NARQ admiration scales were descriptively more strongly correlated with the other D-NARQ admiration scales than with the D-NARQ rivalry scales, and the D-NARQ rivalry scales were descriptively more strongly correlated with the other D-NARQ rivalry scales than with the D-NARQ admiration scales (Table 3). Due to individual differences in propensities to act out narcissistic strivings in one domain rather than in other domains, forms of narcissism in the same domain should be more strongly related to one another than process-specific forms of narcissism in different domains. In line with this notion, the D-NARQ admiration and rivalry scales from the same domain tended to correlate more strongly with each other than the D-NARQ admiration and rivalry scales from different domains (Table 3). Finally, the D-NARQ scales were descriptively more strongly correlated with one another when the domains were closely related to one another (e.g., the two agentic domains of intellectual ability and social dominance) than when the domains were not closely related to one another (e.g., the agentic domain of intellectual ability and the communal domain of communal care; Table 3).

**Table 3.** Intercorrelations Among the D-NARQ Scales.

	INT ADM	DOM ADM	COM ADM	PHY ADM	INT RIV	DOM RIV	COM RIV
DOM ADM	.59						
COM ADM	.45	.49					
PHY ADM	.48	.46	.39				
INT RIV	.59	.37	.18	.33			
DOM RIV	.56	.53	.23	.36	.83		
COM RIV	.42	.33	.40	.30	.69	.64	
PHY RIV	.46	.28	.15	.50	.78	.73	.62

*Note*. *N* = 1,633 to 1,635. The table displays bivariate Pearson product-moment correlations of scale scores (i.e., unweighted average scores). All correlation coefficients in the table were significant at *p* ≤ .001. INT = intellectual ability; PHY = physical attractiveness; DOM = social dominance; COM = Communal Care; ADM = narcissistic admiration; RIV = narcissistic rivalry.

### Convergent and Discriminant Validity

Next, we investigated the convergent and discriminant validity of the D-NARQ by examining the correlations of the D-NARQ scales with established narcissism scales, the Big Five personality traits, and comparative self-evaluations. For several correlations, we tested whether the sizes of two correlation coefficients were significantly different from one another using Steiger’s *Z* test for dependent correlations in the R package cocor (version 1.1-3; Diedenhofen & Musch, 2015; Steiger, 1980). Additionally, we computed double-entry intraclass correlations (e.g., Furr, 2010) to quantify the similarity of the nomological network profiles using the R package iccde (version 0.3.1; Blötner & Grosz, 2021). The double-entry intraclass correlation is a Pearson correlation between two doubly entered profiles. The more positive the double-entry intraclass correlation coefficient is, the more similar the two nomological network profiles are. The most relevant correlation coefficients and profile similarities are presented in Table 4 (for the full correlation matrix, see online Table S14; for profile similarities between all the D-NARQ scales, see online Table S15). In support of its convergent validity, all the D-NARQ admiration scales were more strongly correlated with the NARQ admiration scale (*r*s = .58 to .77) than with the NARQ rivalry scale (*r*s = .11 to .48; *p*s for Steiger’s *Z* test ≤ .001). Vice versa, all the D-NARQ rivalry scales were more strongly correlated with the NARQ rivalry scale (*r*s = .66 to .84) than with the NARQ admiration scale (*r*s = .42 to .54; *p*s for Steiger’s *Z* test ≤ .001). Furthermore, the patterns of correlations of the D-NARQ scales with the NPI and the NPI scales were similar to the pattern found for the two NARQ scales (Table 4; see also Back et al., 2013). That is, the D-NARQ admiration scales were particularly strongly correlated with NPI Leadership/Authority (*r*s = .30 to .75) and/or NPI Grandiose Exhibitionism (*r*s = .18 to .65), whereas the D-NARQ rivalry scales were particularly strongly correlated with NPI Entitlement/Exploitativeness (*r*s = .33 to .52).

**Table 4.** Nomological Networks of the D-NARQ and NARQ Scales.

	Narcissistic admiration					Narcissistic rivalry				
	INT	DOM	COM	PHY	NARQ ADM	INT	DOM	COM	PHY	NARQ RIV
*Narcissism scales*										
NARQ ADM	.77_a_	.73_b_	.58_c_	.63_c_		.49_b_	.54_a_	.42_c_	.44_c_	.41_c_
NARQ RIV	.48_a_	.30_c_	.11_d_	.30_c_	.41_b_	.84_a_	.79_b_	.66_c_	.76_b_	
NPI total	.56_b_	.71_a_	.29_c_	.55_b_	.69_a_	.38_b_	.49_a_	.21_c_	.36_b_	.34_b_
NPI L/A	.46_c_	.75_a_	.30_d_	.32_d_	.55_b_	.28_b_	.42_a_	.14_d_	.19_cd_	.22_c_
NPI GE	.30_c_	.37_c_	.18_d_	.65_a_	.48_b_	.17_b_	.21_b_	.08_c_	.29_a_	.19_b_
NPI E/E	.35_a_	.33_a_	.00_c_	.20_b_	.31_a_	.49_a_	.52_a_	.33_c_	.42_b_	.49_a_
NGS	.65_b_	.64_b_	.49_c_	.54_c_	.74_a_	.36_b_	.44_a_	.28_c_	.34_bc_	.28_c_
CNI	.41_c_	.38_c_	.69_a_	.41_c_	.55_b_	.15_b_	.19_b_	.32_a_	.18_b_	.07_c_
*Big Five personality traits (BFI-S)*										
E	.09_d_	.39_a_	.21_c_	.26_bc_	.30_b_	−.08_bc_	−.03_a_	−.08_bc_	−.05_ab_	−.11_c_
N	−.10_a_	−.26_c_	−.10_a_	−.07_a_	−.20_b_	.11_c_	.01_d_	.17_a_	.11_bc_	.16_ab_
A	−.21_d_	−.18_cd_	.17_a_	−.06_b_	−.13_c_	−.39_b_	−.38_b_	−.22_a_	−.29_a_	−.42_b_
O	.23_a_	.15_b_	.18_ab_	.15_b_	.25_a_	−.05_ab_	−.07_ab_	−.02_a_	−.07_ab_	−.09_b_
C	.00_b_	.14_a_	.14_a_	.05_b_	.04_b_	−.19_b_	−.13_a_	−.10_a_	−.17_ab_	−.21_b_
*Comparative self-evaluations*										
INT	.46_a_	.33_bc_	.26_cd_	.18_d_	.36_b_	.17_a_	.17_a_	.10_ab_	.08_b_	.06_b_
DOM	.37_c_	.71_a_	.27_d_	.27_d_	.45_b_	.12_b_	.27_a_	.04_c_	.05_c_	.04_c_
COM	−.12_c_	−.01_b_	.36_a_	.01_b_	.01_b_	−.35_b_	−.34_b_	−.08_a_	−.32_b_	−.42_c_
PHY	.20_c_	.28_b_	.19_c_	.59_a_	.29_b_	.01_bc_	.06_b_	.01_bc_	.14_a_	−.01_c_
*Profile similarities (rICCs) of D-NARQ scales within the same process dimension*										
DOM	.80					.97				
COM	.50	.38				.86	.81			
PHY	.72	.63	.45			.97	.94	.90		

*Note*. *N*s varied from 1,202 to 1,635 due to pairwise deletion of missing cases. The table presents Pearson product-moment correlation coefficients of scale scores (i.e., unweighted mean scores). Correlation coefficients within the same row and process dimension (e.g., narcissistic admiration) were not significantly different according to Steiger’s *Z* test for dependent correlations if they share a common subscript. To quantify the similarity of the nomological network profiles within the same process dimension, we computed double-entry intraclass correlations (*r*ICCs; e.g., Furr, 2010). D-NARQ = Domain-Specific Narcissistic Admiration and Rivalry Questionnaire; NARQ = Narcissistic Admiration and Rivalry Questionnaire (Domain-Unspecific); ADM = narcissistic admiration; RIV = narcissistic rivalry; NPI = Narcissistic Personality Inventory; L/A = Leadership/Authority; GE = Grandiose Exhibitionism; E/E = Entitlement/Exploitativeness; CNI = Communal Narcissism Inventory; BFI-S = short form of the Big Five Inventory; N = Neuroticism; E = Extraversion; O = Openness to experiences; A = Agreeableness; C = Conscientiousness; INT = intellectual ability; PHY = Physical Attractiveness; DOM = Social Dominance; COM = Communal Care; rICCs = double-entry intraclass correlations.

The convergent and discriminant validities of the D-NARQ were also supported by differences in the nomological networks among the D-NARQ scales, although the differences were more pronounced among the D-NARQ admiration scales (average *r*ICC = .58) than among the D-NARQ rivalry scales (average *r*ICC = .91; Table 4). The social-dominance-specific admiration scale was more strongly correlated with NPI Leadership/Authority (*r* = .75) than the other admiration scales were (*r*s = .30 to .46; *p*s for Steiger’s *Z* test ≤ .001), and the social-dominance-specific rivalry scale was more strongly correlated with Entitlement/Exploitativeness (*r* = .52) than the physical attractiveness and communal care rivalry scales were (*r* = .42 and .33, respectively; both *p*s for Steiger’s *Z* test ≤ .001). Furthermore, the physical-attractiveness-specific admiration scale was more strongly correlated with NPI Grandiose Exhibitionism (*r* = .65) than the other domain-specific admiration scales were (*r*s = .18 to .37; *p*s for Steiger’s *Z* test ≤ .001). Finally, the communal-care-specific admiration scale was more strongly positively correlated with the CNI (*r* = .69) than the other domain-specific admiration scales were (*r*s = .38 to .41; *p*s for Steiger’s *Z* test ≤ .001).

The associations with established narcissism scales supported not only the convergent and discriminant validity of the D-NARQ but also our sorting of established narcissism items according to the process × domain conceptualization of narcissism in Table 1. For example, the NPI total scale seems to measure narcissistic admiration and rivalry, particularly in the social dominance domain (*r* = .71 and .49, respectively), to a lesser extent in the intellectual ability (*r* = .56 and .38, respectively) and physical attractiveness domains (*r* = .55 and .36, respectively), and to the smallest extent in the communal care domain (*r* = .29 and .21, respectively). The low communal-care-specific item content in the NPI is in line with research that contrasts the NPI with communal narcissism measures (e.g., Gebauer, Sedikides, et al., 2012). As opposed to the NPI, the NARQ narcissistic admiration scale and the NGS total scale seem to focus not only on social-dominance-specific but also on intellectual-ability-specific narcissistic admiration (see Table 4).

Next, we examined the correlations between the D-NARQ scales and the Big Five personality traits (Table 4). Just like the admiration scale from the NARQ, the D-NARQ admiration scales were positively correlated with extraversion (*r*s = .09 to .39) and openness (*r*s = .15 to .23) and mostly negatively correlated with neuroticism (*r*s = −.26 to −.07). Like the rivalry scale from the NARQ, most D-NARQ rivalry scales were positively correlated with neuroticism (*r*s = .01 to .17) and negatively correlated with agreeableness (*r*s = −.39 to −.22) and conscientiousness (*r*s =−.19 to −.10). Yet, the strengths and sometimes even the directions of these associations varied across the D-NARQ domains. For example, the intellectual-ability-specific admiration scale had a weaker positive correlation with extraversion (*r* = .09) than the other admiration scales of the D-NARQ did (*r*s = .21 to .39; *p*s for Steiger’s *Z* test ≤ .001). Furthermore, the social-dominance-specific admiration scale had a stronger negative correlation with neuroticism (*r* = −.26) than the other admiration scales from the D-NARQ did (*r*s = −.10 to −.07 ; *p*s for Steiger’s *Z* test ≤ .001), and the social-dominance-specific rivalry scale was less positively correlated with neuroticism (*r* = .01) than the other D-NARQ rivalry scales were (*r*s = .11 to .17). Moreover, the two D-NARQ scales focusing on communal care were less strongly negatively correlated or more strongly positively correlated with agreeableness and conscientiousness than most of the scales from the other domains were (Table 4). In particular, the communal-care-specific admiration scale was positively correlated with agreeableness (*r* = .17), whereas the intellectual-ability-specific and social-dominance-specific admiration scales were negatively correlated with agreeableness (*r* = −.21 and −.18, respectively). Given that social dominance is the domain that is most strongly present in the NPI, and communal care is the domain that is most strongly present in the CNI, these results fit with previous research. For example, Nehrlich et al. (2019) found that the NPI was negatively related to self-perceived prosociality, whereas the CNI was positively related to self-perceived prosociality.

Finally, all D-NARQ admiration scales were more positively correlated with self-evaluations in their own domain than the D-NARQ admiration scales from other domains and the NARQ admiration scale. The intellectual-ability-specific admiration scale was more strongly correlated with comparative self-evaluations regarding intellectual ability (*r* = .46) than the other three D-NARQ admiration scales and the NARQ admiration scale (*r*s = .18 to .36; *p*s for Steiger’s *Z* test ≤ .001). The social-dominance-specific admiration scale was more strongly correlated with comparative self-evaluations regarding social dominance (*r* = .71) than the other three D-NARQ admiration scales and the NARQ admiration scale (*rs* = .27 to .45; *p*s for Steiger’s *Z* test ≤ .001). The communal-care-specific admiration scale was more strongly correlated with comparative self-evaluations regarding communal care (*r* = .36) than the other three D-NARQ admiration scales and the NARQ admiration scale (*r*s = −.12 to .01; *p*s for Steiger’s *Z* test ≤ .001). The physical-attractiveness-specific admiration scale was more strongly correlated with comparative self-evaluations regarding physical attractiveness (*r* = .59) than the other three D-NARQ admiration scales and the NARQ admiration scale (*r*s = .19 to .29; *p*s for Steiger’s *Z* test ≤ .001).

## Discussion

The current study introduced the D-NARQ to integrate two central but yet separate research strands that highlight the multidimensional nature of narcissism in terms of underlying processes and domains. The D-NARQ scales demonstrated acceptable to good psychometric properties in terms of factor structure, measurement precision, and convergent and discriminant validity in a large online study. These initial findings suggest that the two research strands can be integrated and that the two narcissistic processes, narcissistic admiration and rivalry, can be meaningfully distinguished across distinct domains.

Domain-specific forms of narcissistic admiration and rivalry can be thought of as narcissistic traits on a lower level of abstraction than the broad domain-unspecific traits of narcissistic admiration and rivalry (as measured with the NARQ). According to the Brunswik symmetry principle (e.g., Wittmann, 1988), traits should be more strongly associated with behaviors and outcomes that fall on the same level of abstraction than with behaviors and outcomes that are located on higher or lower levels of abstraction. This might explain why domain-specific forms of narcissistic admiration (e.g., intellectual-ability-specific narcissistic admiration) were more strongly associated with domain-specific thoughts (e.g., comparative self-evaluations in the intellectual ability domain) than domain-unspecific narcissistic admiration (Table 4). Accordingly, the D-NARQ might be particularly useful when researchers want to investigate domain-specific behaviors and outcomes.

The evidence for the discriminant validity of the four D-NARQ admiration scales was stronger (i.e., lower intercorrelations and more differences in nomological networks) than the evidence for the discriminant validity of the four D-NARQ rivalry scales (Tables 3, 4, S14, and S15). This pattern of findings suggests that narcissistic admiration processes are more differentiated across domains than narcissistic rivalry processes are differentiated across domains. Hence, there might be a stronger need to distinguish between domain-specific forms of narcissistic admiration than between domain-specific forms of narcissistic rivalry. Similarly, approach motivation seems to be more differentiated across domains than avoidance motivation is (Schönbrodt & Gerstenberg, 2012). Yet, it might be too early to draw the general conclusion that rivalry processes are less domain-specific than admiration processes because we were the first to investigate narcissistic rivalry domains (Table 1), and our choice of content domains was more strongly inspired by previous research on narcissistic admiration than by research on narcissistic rivalry. Narcissistic rivalry might be more strongly differentiated in other domains than the ones included in the current study (e.g., perhaps in domains related to physical self-protection).

Furthermore, the current findings suggest that narcissistic processes unfold differently across domains. The D-NARQ scales showed larger correlations with comparative self-evaluations and more negative correlations with agreeableness in the intellectual ability, social dominance, and physical attractiveness domains than in the communal care domain. We think the domains of intellectual ability, social dominance, and physical attractiveness afford overt self-promotional and other-derogative processes, such as overly positive comparative self-evaluations and disagreeableness. However, overt forms of self-promotion, other-derogation, and disagreeableness run counter to the self-concept and attainment of narcissistic goals in the communal care domain. We think narcissistic self-promotion and other-derogation are still possible in the communal care domain, but these processes might manifest in more covert ways, such as self-promotion via humble bragging or other-derogation via passive aggressiveness. Future research might want to investigate the associations of the D-NARQ scales with covert methods of self-promotion and other-derogation to obtain a better understanding of how narcissistic processes unfold in different domains.

### Limitations and Future Research

In the current study, we proposed a process- and domain-specific conceptualization and assessment of narcissism by introducing the D-NARQ. Although the psychometric soundness of the D-NARQ was largely supported, the current study relied exclusively on cross-sectional self-report data. Previous studies have suggested that the D-NARQ admiration scales are related to overclaiming behavior (Grosz et al., 2017), and the NARQ has been found to be related to a number of actual behaviors and outcomes (e.g., Back et al., 2013; Wurst et al., 2017; for an overview, see Back, 2018). Future research should aim to test whether the D-NARQ scales are uniquely related to behaviors and outcomes in a range of different contexts. For example, are the intellectual-ability-specific D-NARQ scales uniquely linked to self-promotion and other-derogation in academic contexts? Or do people high on physical-attractiveness-specific rivalry react more aggressively to ego threats in the physical attractiveness domain and less aggressively to ego threats in the intellectual ability domain than people high on intellectual-domain-specific rivalry?

Similarly, the intra- and interpersonal causes and consequences of the various domain-specific forms of narcissism could be investigated with the D-NARQ. For example, why do some people employ a self-promotion strategy (narcissistic admiration) in the intellectual ability domain, whereas others self-promote in the communal care domain? Narcissistic self-promotion might be more prevalent in domains in which a person has abilities than in domains in which the person lacks abilities. Alternatively (or additionally), narcissistic people might be more likely to self-promote in the domains that are valued by their peers or societal group than in domains that are not valued by their social environment. Future research could investigate such explanations by investigating individual differences in the D-NARQ along with individual differences in specific abilities (e.g., intelligence tests) and the values that are endorsed by the various social groups that participants are embedded in.

Finally, the D-NARQ focuses on two processes and four domains that have been relevant in narcissism research. Yet, the D-NARQ is by no means exhaustive. For instance, it might be worthwhile to develop additional narcissism items for other domains, such as romantic relationships (e.g., “I am an extraordinarily good romantic partner”; “most people are horrible romantic partners”), parenthood (e.g., “I am an extraordinarily good parent”; “most people are a failure as parents”), and sexuality (“I know exactly how to sexually satisfy a partner”; “other people are boring in bed”; for similar items, see Widman & McNulty, 2010). These domains might be regarded by many people as very central to their self-view, in terms of both enhancing the self and being in competition with and derogating others. Furthermore, it might be worthwhile to assess domain-specific forms of vulnerable narcissism, which entails processes other than narcissistic admiration and rivalry. We encourage future research to use the D-NARQ as a blueprint for developing items for additional domains and processes. We can see three approaches for selecting additional content domains. First, researchers could select domains on the basis of research on narcissism and social evaluations (as we did). Second, researchers could source ideas for additional domains from research on self-attributes and self-concept (e.g., Marsh & O’Neill, 1984; Pelham & Swann, 1989). Third, researchers could try to use existing frameworks and taxonomies to systematically derive and define all relevant domains. For example, the Fundamental-Motives Framework (e.g., Kenrick et al., 2010) might be useful for deriving relevant domains because narcissistic individuals might strive for a grandiose self and superiority over others in terms of satisfying these fundamental human motives (e.g., self-protection, affiliation, mate acquisition).

### Conclusion

Two prior research approaches have pointed out that it is crucial for the conceptualization and measurement of grandiose narcissism to distinguish multiple processes and multiple domains, respectively (e.g., Back et al., 2013; Gebauer, Sedikides, et al., 2012). The current study aimed to integrate the two approaches by introducing the D-NARQ, a measure of narcissistic self-promotion (admiration) and narcissistic other-derogation (rivalry) processes in four domains (intellectual ability, social dominance, communal care, physical attractiveness). Our findings provide a glimpse into the domain-specificity of the narcissistic processes and motivations that future research should be able to unveil further with the help of the D-NARQ. We call for more research using the D-NARQ, as this will foster a more nuanced, systematic, and comprehensive picture of the intra- and interpersonal dynamics surrounding grandiose narcissism.
